# *Keap1* Deficiency Promotes Macrophage M2 Polarization and Protects *β* Cells

**DOI:** 10.1155/jdr/6786708

**Published:** 2026-10-12

**Authors:** Baoliang Huang, Lunpan Mou, Xiaoyan Huang, Siyu Su, Zhenzhen Hong, Yi Zhang

**Affiliations:** ^1^ Quanzhou First Hospital Affiliated to Fujian Medical University Quanzhou Fujian China fjmu.edu.cn; ^2^ The 910th Hospital of the Joint Logistics Support Force of the Chinese People′s Liberation Army Quanzhou Fujian China

**Keywords:** inflammation, Keap1-Nrf2-ARE pathway, macrophage polarization, NF-*κ*B pathway, oxidative stress, pancreatic *β* cells, Type 2 diabetes mellitus

## Abstract

**Background:**

Progressive pancreatic *β*‐cell injury is a core feature of Type 2 diabetes mellitus (T2DM). Imbalance between macrophage M1 and M2 polarization, driven by chronic inflammation, is a key contributor to this injury. The Keap1‐Nrf2‐ARE pathway regulates macrophage polarization and inflammatory responses, but whether it affects pancreatic *β*‐cell survival through macrophages is unknown.

**Methods:**

We generated a *Keap1*‐knockout THP‐1 line using CRISPR‐Cas9, differentiated the cells into M0 macrophages with PMA, polarized them toward M2 with IL‐4/IL‐13, and cocultured them with MIN6 cells injured by LPS.

**Results:**

*Keap1* knockout increased M2 polarization efficiency and reduced the nuclear‐to‐cytoplasmic ratio of NF‐*κ*B p65, with lower IL‐1*β* and TNF‐*α* mRNA expression. After coculture with LPS‐injured MIN6 cells, the Keap1‐KO‐M2 group showed higher MIN6 viability and less apoptosis. The antiapoptotic protein Bcl‐2 rose, whereas the proapoptotic factors Bax and cleaved‐caspase3 declined; MDA content fell, whereas HO‐1 and SOD levels climbed.

**Conclusion:**

These findings indicate that *Keap1* deficiency attenuates LPS‐induced pancreatic *β*‐cell injury by increasing macrophage M2 polarization efficiency and expanding the M2 population.

## 1. Introduction

Type 2 diabetes mellitus (T2DM) now ranks among the most pressing public health challenges worldwide. Its defining features are the gradual failure of the pancreatic *β*‐cell compartment together with systemic insulin resistance [1]. Within the islet microenvironment, low‐grade persistent inflammation precedes overt *β*‐cell loss, and aberrant activation of resident immune cells helps to establish this state [2–4]. Macrophages dominate the islet immune compartment and retain marked plasticity; their polarization state dictates how the inflammatory response unfolds: M1 macrophages release tumor necrosis factor‐*α* (TNF‐*α*) and interleukin‐1*β* (IL‐1*β*) and aggravate tissue damage, whereas M2 cells liberate anti‐inflammatory mediators that support tissue repair and help preserve homeostasis [5–7]. In T2DM, this equilibrium tilts [8, 9], with the M1 compartment expanded [10]. The resulting surge in proinflammatory mediators drives local accumulation of reactive oxygen species (ROS) within islets and sets oxidative stress in motion. Inflammation and oxidative stress feed on each other, forming a loop that drives *β*‐cell apoptosis, blunts proliferative capacity, and undermines insulin output [11]. Restoring the polarization equilibrium while reining in inflammation and oxidative stress may therefore offer a route to preserving *β*‐cell function and slowing T2DM progression [12].

By coupling redox sensing to transcriptional output, the Keap1‐Nrf2‐ARE pathway governs intracellular redox balance and inflammatory responses [13] and mounts the frontline cellular defense against oxidative and inflammatory insult [14]. At steady state, Kelch‐like ECH‐associated protein 1 (Keap1) sequesters Nrf2 and channels it toward ubiquitination, holding Nrf2 abundance low [15, 16]. Oxidative or inflammatory cues release this brake, permitting Nrf2 to accumulate within the nucleus and switch on genes encoding antioxidant enzymes—including heme oxygenase‐1 (HO‐1) and superoxide dismutase (SOD)—alongside anti‐inflammatory effectors, together curtailing cell injury [14]. The same axis has also been implicated in setting macrophage polarization. Boosting Nrf2 activity, whether pharmacologically or by manipulating its expression, shifts macrophages toward the M2 program and dampens proinflammatory mediator release [17, 18]. Kobayashi et al. [18] showed in myeloid cell–specific *Keap1*‐knockout mice that *Keap1* deficiency suppresses proinflammatory cytokine transcription in macrophages through Nrf2 activation. This provided genetic evidence for targeting the macrophage Keap1‐Nrf2 pathway in inflammatory disease. Macrophage infiltration of the islet microenvironment is nevertheless a characteristic alteration in T2DM [4, 9]. Whether macrophages lacking Keap1 can shield *β* cells by reinforcing the M2 program and restraining NF‐*κ*B remains untested. Nor is it clear how Keap1‐Nrf2 and NF‐*κ*B communicate during polarization, or what that means for *β*‐cell fate.

We hypothesized that *Keap1 knockout* promotes macrophage M2 polarization and inhibits NF‐*κ*B signaling. This would reduce proinflammatory factor release, suppress local islet inflammation and oxidative stress, and attenuate lipopolysaccharide (LPS)–induced *β*‐cell injury. To test this, we generated a *Keap1*‐knockout line from THP‐1 cells and examined macrophage polarization, inflammatory cytokines, and NF‐*κ*B nuclear translocation. We then cocultured *Keap1*‐knockout M2 macrophages with LPS‐injured MIN6 cells and assessed *β*‐cell viability, apoptosis, inflammation, and oxidative stress.

## 2. Materials and Methods

### 2.1. Cell Culture and Stimulation

THP‐1, a human monocytic cell line, was purchased from Wuhan Procell Life Science & Technology Co. Ltd. (Wuhan, Hubei, China) and tested negative for mycoplasma contamination before use. THP‐1 monocytes were kept at 37°C in a humidified 5% CO_2_ incubator, in THP‐1 cell‐specific medium (CM‐0233, Wuhan Procell Life Science & Technology Co. Ltd., Wuhan, Hubei, China) supplemented with 1% penicillin‐streptomycin (P1400, Beijing Solarbio Science & Technology Co. Ltd., Beijing, China). To push the cells toward an M0 macrophage state, medium was supplemented with 100‐ng/mL phorbol 12‐myristate 13‐acetate (PMA) (HY‐18739, MedChemExpress LLC, Monmouth Junction, New Jersey, United States) and left on the cells for 48 h. M0 macrophages were subsequently skewed toward the M2 phenotype by a further 48 h in PMA‐free fresh medium containing 20 ng/mL IL‐4 (CX03, Novoprotein Scientific Inc., Shanghai, China) and 20 ng/mL IL‐13 (CC89, Novoprotein Scientific Inc., Shanghai, China). Cells receiving PMA alone, without IL‐4/IL‐13, served as M0 macrophage controls.

### 2.2. *Keap1* Gene Knockout

Following previously reported CRISPR/Cas9 knockout protocols for THP‐1 cells [19, 20], a *Keap1*‐specific sgRNA sequence (CCTCATTGAATTCGCCTACACGG) was designed with the Chopchop online sgRNA design system (https://chopchop.cbu.uib.no/). The sgRNA was cloned into the lenti‐Cas9 vector, which was then cotransfected into HEK293T cells together with the Gag‐Pol and pCMV‐VSVG packaging plasmids for lentivirus production. The harvested lentiviral particles were used to transduce THP‐1 cells. After 48 h, the cells were switched to fresh complete medium containing puromycin and selected for 48–72 h, by which point every uninfected control cell had died. The remaining cells were rinsed twice with PBS, replated in fresh complete medium, and passaged repeatedly until a stable *Keap1*‐knockout line was obtained (THP‐1 Keap1‐KO). Knockout efficiency was verified by Western blot detection of Keap1 protein expression. An empty vector‐transfected group (THP‐1 Ctrl) and a wild‐type group (THP‐1 WT) served as controls.

### 2.3. Flow Cytometry

Macrophages from each group were collected and fixed with fixation buffer (00‐8222‐49, Invitrogen [Thermo Fisher Scientific], Carlsbad, California, United States) for 20–60 min protected from light. After a 5‐min spin at 400 g at room temperature, pelleted cells were taken up in permeabilization buffer (00‐8333‐56, Invitrogen [Thermo Fisher Scientific], Carlsbad, California, United States) and the supernatant was removed. Cells were then stained for 20–60 min in the dark with fluorochrome‐conjugated antibodies against CD11b (340007), CD68 (333805), CD206 (321104), and CD163 (333606), all purchased from BioLegend (San Diego, California, United States). Events were captured on a NovoCyte flow cytometer (NovoCyte 2060R, Agilent Biosciences [Hangzhou] Co. Ltd., Hangzhou, Zhejiang, China), and surface marker levels were analyzed in FlowJo v10.8.1 (FlowJo, LLC, Ashland, Oregon, United States).

Apoptosis in MIN6 cells was assessed after detachment with EDTA‐free trypsin and three rinses in ice‐cold PBS. Pellets were taken up in 100 *μ*L PBS, to which 5‐*μ*L Annexin V‐FITC was added together with 10 *μ*L PI (AP101‐100‐kit, Hangzhou MultiSciences Biotech Co. Ltd. [Multi Sciences], Hangzhou, Zhejiang, China); the tubes were left at room temperature for 15 min in the dark. The apoptotic fraction was then determined on a NovoCyte flow cytometer.

### 2.4. RT‐qPCR

RNA was extracted from THP‐1 macrophages and from cocultured MIN6 cells using an ultrapure RNA kit (CW0581M, Beijing CWBIO Biotechnology Co. Ltd., Beijing, China); its yield and purity were checked on a UV spectrophotometer (NP80, Implen GmbH, Munich, Germany). cDNA was synthesized using HiScript II Q RT SuperMix for qPCR (+gDNA wiper) (R223‐01, Nanjing Vazyme Biotech Co. Ltd., Nanjing, Jiangsu, China), and amplification was performed with SuperStar Universal SYBR Master Mix (CW3360M, Beijing CWBIO Biotechnology Co. Ltd., Beijing, China) on a CFXConnect real‐time quantitative PCR system (Bio‐Rad Laboratories, Hercules, California, United States). Gene‐specific primers were used to amplify IL‐1*β*, interleukin‐1*α* (IL‐1*α*), TNF‐*α*, *Keap1*, and *β*‐actin, with the sequences provided in Table 1.

**Table 1:** RT‐qPCR primer sequences.

Target gene	Forward primer (5 ^′^–3 ^′^)	Reverse primer (5 ^′^–3 ^′^)
*β*‐actin	TGGCACCCAGCACAATGAA	CTAAGTCATAGTCCGCCTAGAAGCA
IL‐1*α*	ATTGTATGTGACTGCCCAAGATG	CGTGAGTTTCCCAGAAGAAGAG
TNF‐*α*	CGAGTGACAAGCCTGTAGCC	TGAAGAGGACCTGGGAGTAGAT
IL‐1*β*	GCACGATGCACCTGTACGAT	TGGAGAACACCACTTGTTGC
Keap1	AGAGGAACGAGTGGCGAATG	ACCATCATAGCCTCCAAGGAC

*Note:* Primers were synthesized by General Biosystems (Anhui) Co. Ltd. (Chuzhou, Anhui, China).

Transcript abundance was compared across groups by the 2^−*ΔΔ*Ct^ approach, using *β*‐actin as the internal reference to correct for between‐sample variation. Each sample was run in three independent qPCR reactions and averaged before further analysis.

### 2.5. Immunofluorescence Staining

Macrophages were plated as THP‐1 WT, THP‐1 Ctrl, and THP‐1 Keap1‐KO groups. Cultures were rinsed three times in PBS, fixed for 15 min with 4% paraformaldehyde fixative (P1110, Beijing Solarbio Science & Technology Co. Ltd., Beijing, China), and rinsed again three times in PBS. Permeabilization was carried out with 0.5% (*v*/*v*) Triton X‐100 in PBS; after three further PBS rinses, nonspecific sites were blocked with 5% BSA (A8020, Beijing Solarbio Science & Technology Co. Ltd., Beijing, China) for 1 h at 37°C. Cells were exposed overnight at 4°C to a rabbit anti‐NF‐*κ*B p65 primary antibody (AF5006, Affinity Biosciences, Liyang, Jiangsu, China, 1:200), rinsed three times in PBS, and then held for 35 min at 37°C with a Cy3‐conjugated goat anti‐rabbit IgG (H + L) secondary antibody (AS007, Wuhan ABclonal Biotechnology Co. Ltd., Wuhan, Hubei, China, 1:200), followed by thorough rinsing in PBS. DAPI (KGA1808‐50, Nanjing KeyGEN Biotech Co. Ltd., Nanjing, Jiangsu, China) was applied for 2 min to counterstain nuclei; excess dye was removed with PBS and slides were mounted in 50% glycerol. Images were captured on a fluorescence microscope (CKX53, Olympus Corporation, Tokyo, Japan). DAPI (excitation 330–380 nm, emission 420 nm) generated the blue nuclear signal, whereas Cy3 (excitation 510–560 nm, emission 590 nm) produced the red signal identifying NF‐*κ*B (p65). ImageJ (National Institutes of Health, Bethesda, Maryland, United States) was used to compute the nuclear‐to‐cytoplasmic p65 ratio from mean fluorescence intensity recorded separately over the nuclear compartment (DAPI‐positive area) and the cytoplasm. Three independent experiments were run per group, with at least three random fields imaged per well. Statistically, the average across all fields of one independent experiment counted as a single observation, so the three experiments constituted the statistical unit (*n* = 3); technical replicates were therefore not treated as biological replicates.

### 2.6. Coculture of THP‐1 Macrophages and MIN6 Cells

The mouse pancreatic *β*‐cell line MIN6 (FH0390, Shanghai Fuheng Biotechnology Co. Ltd., Shanghai, China), which arose from an SV40 large T antigen transgenic C57BL/6 mouse insulinoma and retains glucose‐inducible insulin secretion [21], was challenged for 24 h with LPS (HY‐D1056, MedChemExpress LLC, Monmouth Junction, New Jersey, United States) at 100 ng/mL to model inflammatory injury. The LPS‐containing medium was then removed and replaced with fresh medium without LPS. Coculture was carried out in Transwell inserts: MIN6 cells occupied the lower compartment, whereas macrophages (M0 or M2) of each group were placed in the upper compartment at a 5:1 macrophage‐to‐MIN6 ratio for 24 h. The lower chamber MIN6 cells were then harvested for assessment of viability, apoptosis, inflammatory cytokines, and oxidative stress indicators.

### 2.7. Cell Viability (CCK‐8 Assay)

Once coculture ended, MIN6 cultures in 96‐well plates were fed 100‐*μ*L fresh medium per well before addition of 10‐*μ*L CCK‐8 reagent (KGA317, Nanjing KeyGEN Biotech Co. Ltd., Nanjing, Jiangsu, China). Plates were returned to 37°C for 2 h, after which absorbance at 450 nm was recorded on a microplate reader (WD‐2012B, Beijing Liuyi Biotechnology Co. Ltd., Beijing, China). Since absorbance tracks the metabolic activity of living cells, viability is reported as relative absorbance per well.

### 2.8. Western Blotting

Cells from each group were harvested and lysed on ice for 20 min in RIPA cell lysis buffer (C1053, Beijing Pulilai Gene Technology Co. Ltd., Beijing, China), then spun at 12,000 r/min for 10 min at 4°C. Cleared lysates were used for protein quantification with a BCA protein assay kit (E‐BC‐K318‐M, Wuhan Elabscience Biotechnology Co. Ltd., Wuhan, Hubei, China). Equal protein loads were separated by 10% sodium dodecyl sulfate‐polyacrylamide gel electrophoresis (SDS‐PAGE) and blotted onto PVDF membranes (IPVH00010, MilliporeSigma, Burlington, Massachusetts, United States). Membranes were blocked with 5% skim milk for 1 h at room temperature and then probed overnight at 4°C using primary antibodies against Keap1 (rabbit anti‐Keap1, AF5266, Affinity Biosciences, Liyang, Jiangsu, China, 1/1000), Bcl2 (rabbit anti‐Bcl2, AF6138, Affinity Biosciences, Liyang, Jiangsu, China, 1:1000), Bax (rabbit anti‐Bax, 50599‐2‐Ig, Proteintech Group, Inc., Rosemont, Illinois, United States, 1:2000), cleaved‐caspase3 (rabbit anti‐cleaved‐caspase3, AF7022, Affinity Biosciences, Liyang, Jiangsu, China, 1:1000), and GAPDH (mouse anti‐GAPDH, HC301, Beijing TransGen Biotech Co. Ltd., Beijing, China, 1:2000). Three 10‐min TBST washes preceded a 2‐h incubation at room temperature with HRP‐conjugated secondary antibodies (goat anti‐mouse IgG (H + L), GB23301, Wuhan Servicebio Technology Co. Ltd., Wuhan, Hubei, China, 1:2000; goat anti‐rabbit IgG (H + L), GB23303, Wuhan Servicebio Technology Co. Ltd., Wuhan, Hubei, China, 1:2000), after which the membranes received three further TBST washes of 10 min each. Protein bands were developed by applying enhanced chemiluminescence substrate (RJ239676, Thermo Fisher Scientific, Waltham, Massachusetts, United States) and captured with a fully automated chemiluminescence imaging system (Tanon‐5200, Shanghai Tanon Science & Technology Co. Ltd., Shanghai, China). Band intensity was measured in ImageJ, and each target protein was normalized to GAPDH. For Figure 1B, THP‐1 WT was set as the reference; for the coculture experiments, the MIN6‐only culture group served as the reference, with the relative target protein value set to 1.

**Figure 1:**
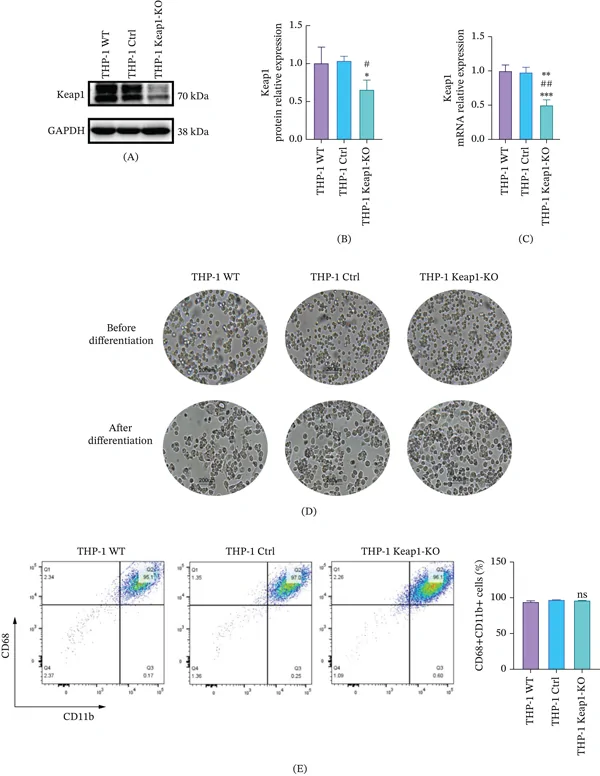
(A,B) Western blot verification of Keap1 protein expression and its quantitative analysis, (C) qPCR verification of *Keap1* gene expression, (D) observation of cell morphology, and (E) flow cytometric analysis of CD68 and CD11b expression. Data are presented as mean ± SD (*n* = 3 independent experiments). ∗ indicates comparison between the Keap1‐KO group and the WT group, and # indicates comparison between the Keap1‐KO group and the Ctrl group. ∗or #p < 0.05, ∗∗ or ##p < 0.01 , ∗∗∗ or ###p < 0.001; ns indicates no statistically significant difference. ∗ = Keap1‐KO versus WT; # = Keap1‐KO versus Ctrl. Panel (B) shows # and ∗; panel C shows ## and ∗∗∗.

### 2.9. Measurement of Oxidative Stress–Related Indicators

After coculture, MIN6 cells were collected and spun at 4°C for 10 min, after which the supernatants were harvested. Levels of malondialdehyde (MDA), HO‐1, and SOD were determined with commercial kits, namely a MDA assay kit (E‐EL‐0060, Wuhan Elabscience Biotechnology Co. Ltd. [Elabscience], Wuhan, Hubei, China), a HO‐1 assay kit (MM‐1508H2, Shanghai Enzyme‐linked Biotechnology Co. Ltd., Shanghai, China), and an SOD assay kit (A001‐3‐2, Nanjing Jiancheng Bioengineering Institute, Nanjing, Jiangsu, China). All assays were performed following the manufacturers′ instructions.

### 2.10. Statistical Analysis

Every experiment was carried out on three independent occasions. Statistical analysis and graphing were done with GraphPad Prism 10.0 (GraphPad Software, San Diego, California, United States). Quantitative data are reported as mean ± SD (standard deviation). Two‐group comparisons were assessed by independent‐sample *t*‐tests, and comparisons across three or more groups by one‐way ANOVA. The two‐factor design comprising macrophage genotype and polarization state, along with the M2 polarization time‐course data, was analyzed by two‐way ANOVA. Where ANOVA was significant, Tukey′s post hoc test was used for pairwise comparisons. Significance thresholds were ∗*p* < 0.05, ∗∗*p* < 0.01, ∗∗∗*p* < 0.001, and ∗∗∗∗*p* < 0.0001, with ns marking differences that did not reach significance (*p* > 0.05).

## 3. Results

### 3.1. Verification of *Keap1* Gene Knockout and Macrophage Differentiation

We generated a *Keap1*‐knockout THP‐1 line (THP‐1 Keap1‐KO) using CRISPR‐Cas9, with a wild‐type control (THP‐1 WT) and an empty vector control (THP‐1 Ctrl). Knockout efficiency was verified by qPCR (Figure 1C) and Western blot (Figure 1A,B). Compared with both controls, *Keap1* mRNA expression in the THP‐1 Keap1‐KO group was significantly lower (Figure 1C; *p* < 0.001 vs. WT, *p* < 0.01 vs. Ctrl), and protein levels were reduced (Figure 1A,B; *p* < 0.05). Knockout was therefore successful.

Following a 48‐h induction with 100 ng/mL PMA, THP‐1 cells became adherent and changed from a round to an enlarged, irregular shape with loose cytoplasm (Figure 1D). Flow cytometry showed that more than 90% of cells were CD68 + CD11b+ (Figure 1E), confirming differentiation into M0 macrophages. THP‐1 Keap1‐KO cells showed no difference from the WT or Ctrl groups in growth state or morphology after the same induction. The proportion of CD68 + CD11b+ cells also remained above 90% (*p* > 0.05), indicating that *Keap1* knockout does not affect differentiation into M0 macrophages.

### 3.2. *Keap1* Gene Knockout Promotes Macrophage M2 Polarization

To induce M2 polarization, M0 THP‐1 macrophages were exposed for 48 h to 20 ng/mL IL‐4 plus 20 ng/mL IL‐13. CD163 and CD206, two M2‐associated markers, were tracked by flow cytometry at 0, 2, 8, 24, and 48 h (Figure 2A). In the THP‐1 WT group (WT‐M*φ*) and the THP‐1 Ctrl group (vector‐M*φ*), the proportion of CD206 + CD163+ double‐positive cells rose slowly with time, reaching approximately 13% at 48 h. In the THP‐1 Keap1‐KO group (Keap1‐KO‐M*φ*), the proportion rose to 31.54% by 8 h and 55.27% by 48 h (Figure 2A,B; *p* < 0.001). *Keap1* knockout therefore promotes M2 polarization of THP‐1 macrophages.

**Figure 2:**
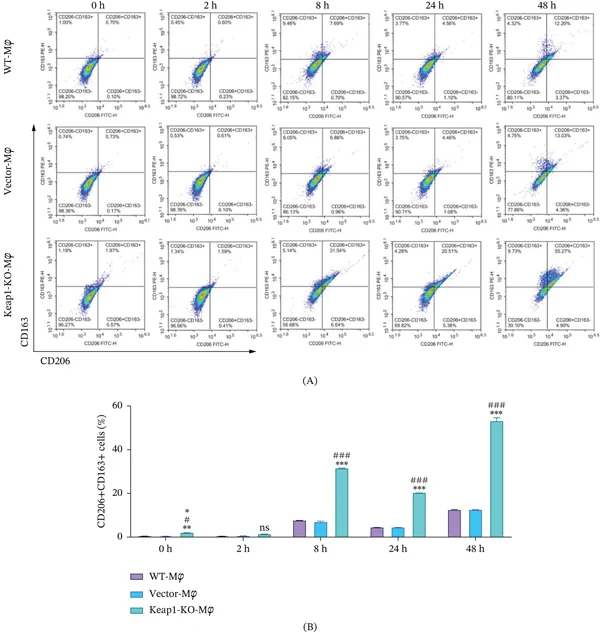
(A) Representative scatter plots of flow cytometric detection of CD206/CD163 expression at each time point (0, 2, 8, 24, and 48 h); (B) quantitative analysis of the proportion of CD206 + CD163+ double‐positive cells (double positive refers to cells expressing both CD206 and CD163). Data are presented as mean ± SD (*n* = 3 independent experiments). ∗ indicates comparison between the Keap1‐KO‐M*φ* group and the WT‐M*φ* group; # indicates comparison between the Keap1‐KO‐M*φ* group and the vector‐M*φ* group;  ^∗^or #p < 0.05,  ^∗∗^ or ##p < 0.01 ,  ^∗∗∗^ or ###p < 0.001; ns indicates no statistically significant difference.  ^∗^ = Keap1‐KO‐M*φ* versus WT‐M*φ*; # = Keap1‐KO‐M*φ* versus vector‐M*φ*. In Panel (B), 0 h shows # and  ^∗∗^; 2 h shows ns; and 8, 24 and 48 h show ### and  ^∗∗∗^.

### 3.3. *Keap1* Deficiency Is Accompanied by Reduced NF‐*κ*B p65 Nuclear‐to‐Cytoplasmic Ratio and Decreased Inflammatory Cytokine Expression

Immunofluorescence staining (DAPI/p65) was used to track the subcellular localization of p65 during M2 polarization (0, 2, 8, 24, and 48 h) in the WT‐M*φ*, vector‐M*φ*, and Keap1‐KO‐M*φ* groups (Figure 3A), and the p65 nuclear‐to‐cytoplasmic ratio was quantified (Figure 3B). Nuclear translocation of the p65 subunit is a key marker of NF‐*κ*B activation [22]. In the WT‐M*φ* and vector‐M*φ* groups, p65 fluorescence overlapped extensively with the nuclei. In the Keap1‐KO‐M*φ* group, p65 was predominantly retained in the cytoplasm. The three groups did not differ at 0 and 2 h. With prolonged polarization, the ratio rose in the control groups but remained significantly lower in the Keap1‐KO‐M*φ* group from 8 h onward (8, 24, and 48 h; *p* < 0.001). qPCR confirmed lower mRNA expression of the NF‐*κ*B–dependent proinflammatory cytokines IL‐1*β* and TNF‐*α* in the Keap1‐KO‐M*φ* group (*p* < 0.001; Figure 3C,D). In the context of M2 polarization, *Keap1* deficiency therefore suppresses NF‐*κ*B p65 nuclear translocation and the proinflammatory transcriptional program it mediates. This inhibition began at 8 h. That timing coincides with the window required for Nrf2 downstream antioxidant and anti‐inflammatory target genes such as HO‐1 to be transcribed, translated, and become functional. The negative regulation of NF‐*κ*B by *Keap1* deficiency may therefore depend on prior establishment of a functional antioxidant and anti‐inflammatory program.

**Figure 3:**
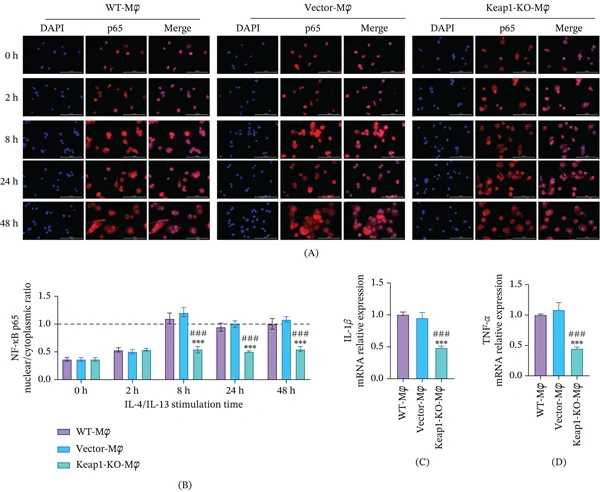
(A) Representative images of immunofluorescence detection of NF‐*κ*B p65 subunit nuclear localization (DAPI labels nuclei, Cy3 labels p65); (B) quantitative analysis of the p65 nuclear‐to‐cytoplasmic ratio (*n* = 3 independent experiments, with at least three random fields averaged per sample; mean ± SD; differences among the three groups at 0 and 2 h were not statistically significant, whereas the Keap1‐KO‐M*φ* group was significantly lower than the WT‐M*φ* and vector‐M*φ* groups at 8, 24, and 48 h, *p* < 0.001); (C,D) qPCR detection of IL‐1*β* and TNF‐*α* mRNA expression levels in M2 macrophages. Data are presented as mean ± SD (*n* = 3 independent experiments). ∗ indicates comparison between the Keap1‐KO‐M*φ* group and the WT‐M*φ* group; # indicates comparison between the Keap1‐KO‐M*φ* group and the vector‐M*φ* group; ∗*p* < 0.05, ∗∗*p* < 0.01, ∗∗∗*p* < 0.001; ns indicates no statistically significant difference. ∗ = Keap1‐KO‐M*φ* versus WT‐M*φ*; # = Keap1‐KO‐M*φ* versus vector‐M*φ*. Figure 3 shows ### and ∗∗∗, in Panel (B) and in Panels (C) and (D).

### 3.4. Keap1‐KO‐M2 Macrophages Increase the Viability of LPS‐Injured MIN6 Cells

MIN6 cells with LPS‐pretreatment (100 ng/mL, 24 h)–induced inflammatory injury were cocultured for 24 h with WT‐M*φ*, WT‐M2‐M*φ*, Keap1‐KO‐M*φ*, and Keap1‐KO‐M2‐M*φ* macrophages, with an LPS‐injured MIN6‐only culture as control. Viability was measured by CCK‐8. Compared with the MIN6‐only group, the WT‐M*φ* coculture group showed no change in viability, whereas the WT‐M2‐M*φ* coculture group showed significantly higher viability (Figure 4G; *p* < 0.05). The WT‐M2‐M*φ* group also outperformed the WT‐M*φ* group (*p* < 0.001), and the Keap1‐KO‐M2‐M*φ* group surpassed both the Keap1‐KO‐M*φ* group (*p* < 0.001) and the WT‐M2‐M*φ* group (*p* < 0.01) (Figure 4G). M2 macrophages therefore improve the viability of LPS‐injured MIN6 cells. *Keap1*‐knockout M0 macrophages produced a mild improvement, which was further enhanced after M2 polarization. Because viability was significantly greater in the Keap1‐KO‐M2‐M*φ* group than in the WT‐M2‐M*φ* group (*p* < 0.01), *Keap1* deficiency appears to further enhance macrophage protection of injured MIN6 cell viability beyond M2 polarization alone.

Figure 4(A–E) Representative scatter plots of flow cytometric detection of MIN6 cell apoptosis ((A) MIN6‐only culture; (B) WT‐M*φ* coculture; (C) WT‐M2‐M*φ* coculture; (D) Keap1‐KO‐M*φ* coculture; (E) Keap1‐KO‐M2‐M*φ* coculture). MIN6 cells were pretreated with LPS (100 ng/mL, 24 h) before coculture, and the LPS‐containing medium was replaced with fresh medium before the macrophages were added; (F) quantitative analysis of the apoptosis rate; (G) CCK‐8 detection of MIN6 cell viability; (H) qPCR detection of IL‐1*α* mRNA expression; (I) Western blot detection of Bcl‐2, Bax, and cleaved‐caspase3 protein expression in MIN6 cells (MIN6‐only culture, WT‐M*φ* coculture, WT‐M2‐M*φ* coculture, Keap1‐KO‐M*φ* coculture, Keap1‐KO‐M2‐M*φ* coculture); (J–L) quantitative analysis of Bcl‐2, Bax, and cleaved‐caspase3 protein expression; (M–O) kit‐based measurement of MDA, HO‐1, and SOD levels in MIN6 cells. Data are presented as mean ± SD (*n* = 3 independent experiments). ∗ indicates comparison between the corresponding two groups shown in the figure; ∗*p* < 0.05, ∗∗*p* < 0.01, ∗∗∗*p* < 0.001, ∗∗∗∗*p* < 0.0001; ns indicates no statistically significant difference.

**Figure:**
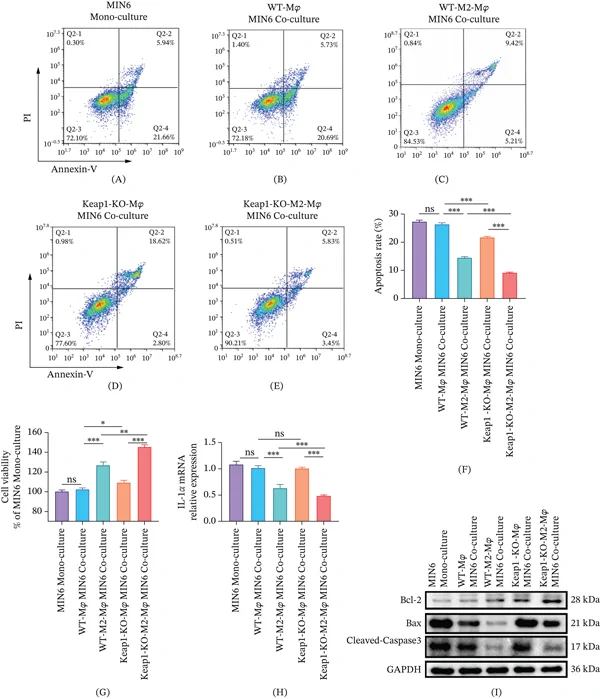

**Figure:**
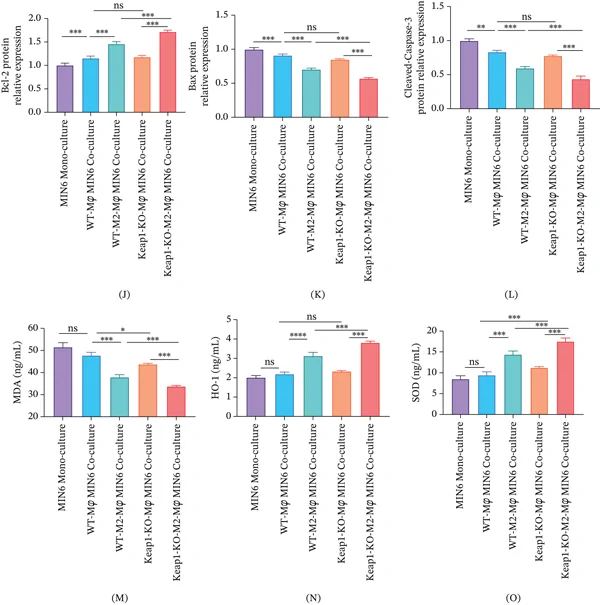

### 3.5. Keap1‐KO‐M2 Macrophages Regulate Apoptosis‐Related Proteins and Proinflammatory Cytokine Expression in MIN6 Cells

MIN6 cell survival and death were evaluated by Annexin V/PI staining with flow cytometric readout. In the MIN6‐only group, 72.10% of cells were viable (Q2–3) and 27.60% were apoptotic (late plus early apoptosis). The WT‐M*φ* coculture group showed 72.18% viable cells and 26.42% apoptotic cells, with no difference from the MIN6‐only group. In the WT‐M2‐M*φ* coculture group, viability rose to 84.53% and apoptosis fell to 14.63%. The Keap1‐KO‐M*φ* coculture group showed 77.60% viable cells and 21.42% apoptotic cells, of which 18.62% were late apoptotic. In the Keap1‐KO‐M2‐M*φ* coculture group, viability rose to 90.21% and apoptosis fell to 9.28%, with late apoptosis at 5.83% (Figure 4A–E). Quantification of the apoptosis rate followed the same trend (Figure 4F). Western blot (Figure 4I) showed that, compared with the Keap1 − KO − *M* *φ* + MIN6 group, MIN6 cells in the Keap1 − KO − M2 − *M* *φ* + MIN6 group had higher antiapoptotic Bcl‐2 expression (Figure 4J; *p* < 0.001) and lower proapoptotic Bax (Figure 4K; *p* < 0.001) and cleaved‐caspase3 (Figure 4L; *p* < 0.001) expression. qPCR showed lower mRNA expression of the proinflammatory cytokine IL‐1*α* in MIN6 cells after coculture with Keap1‐KO‐M2‐M*φ* macrophages (Figure 4H; *p* < 0.001). Relative to the WT‐M2‐M*φ* group, the Keap1‐KO‐M2‐M*φ* coculture showed a lower MIN6 apoptosis rate (Figure 4F; *p* < 0.001), reduced IL‐1*α* mRNA expression (Figure 4H; *p* < 0.001), decreased Bax (Figure 4K; *p* < 0.001) and cleaved‐caspase3 (Figure 4L; *p* < 0.001) expression, and higher Bcl‐2 expression (Figure 4J; *p* < 0.001). Beyond M2 polarization alone, *Keap1* deficiency therefore further enhances the antiapoptotic and anti‐inflammatory protection that macrophages provide to LPS‐injured MIN6 cells.

### 3.6. Keap1‐KO‐M2 Macrophages Ameliorate Oxidative Stress in MIN6 Cells

Oxidative stress indices were measured in MIN6 cells after coculture, including the lipid peroxidation product MDA and the antioxidants HO‐1 and SOD. Relative to the MIN6‐only group, the WT‐M*φ* coculture group showed no change in MDA content, SOD activity, or HO‐1 level (*p* > 0.05; Figure 4M–O). Shifting from WT‐M*φ* to WT‐M2‐M*φ* lowered MDA content (Figure 4M; *p* < 0.001) and raised HO‐1 level (Figure 4N; *p* < 0.0001) and SOD activity (Figure 4O; *p* < 0.001). Substituting Keap1‐KO‐M2‐M*φ* for Keap1‐KO‐M*φ* in the MIN6 coculture further reduced MDA content (Figure 4M; *p* < 0.001) and further increased SOD activity (Figure 4O; *p* < 0.001) and HO‐1 level (Figure 4N; *p* < 0.001). Against the WT‐M2‐M*φ* group, the Keap1‐KO‐M2‐M*φ* group again showed less MDA (Figure 4M; *p* < 0.001) together with greater SOD activity (Figure 4O; *p* < 0.001) and HO‐1 level (Figure 4N; *p* < 0.001). Coculture with Keap1‐KO‐M2 macrophages therefore raised antioxidant molecule expression and suppressed lipid peroxidation in LPS‐injured MIN6 cells. Because HO‐1 and SOD were measured in MIN6 cells, their increase reflects enhanced antioxidant responses of the *β* cells themselves.

## 4. Discussion

How macrophages polarize is a pivotal determinant of the islet inflammatory microenvironment and is tightly bound up with metabolic diseases such as T2DM [23]. Because the Keap1‐Nrf2 pathway sits at the hub of cellular redox homeostasis and inflammatory control, it has been proposed as a target for modulating immune cell function [13]. Although myeloid cell–specific *Keap1* knockout suppresses proinflammatory cytokine transcription in macrophages via Nrf2 activation [18], whether this effect operates within the islet microenvironment and protects *β* cells against inflammatory injury has not been tested. To address this, we established *Keap1*‐knockout (Keap1‐KO) THP‐1 cells with CRISPR‐Cas9 and employed a macrophage‐*β*‐cell Transwell coculture system, evaluating polarization phenotype, inflammatory signaling, *β*‐cell fate, and oxidative stress.

*Keap1* knockout drove M2 polarization in THP‐1–derived macrophages: After 48 h of IL‐4/IL‐13 induction, 55.27% of cells in the Keap1‐KO group were CD206 + CD163+ double‐positive, in line with the reported function of the Keap1‐Nrf2 pathway in macrophage polarization [13, 18]. Under basal conditions, Keap1 keeps Nrf2 levels low by promoting its ubiquitination and degradation [24]. Loss of Keap1 lifts this restraint, permitting Nrf2 to build up and move into the nucleus, where it switches on downstream antioxidant and anti‐inflammatory target genes [13]. In our coculture system, HO‐1 and SOD levels in MIN6 cells increased in parallel with the rise in M2 markers, consistent with Nrf2 pathway activation [25].

From 8 to 48 h of M2 polarization, the Keap1‐KO group displayed a lower p65 nuclear‐to‐cytoplasmic ratio than the control groups, together with reduced IL‐1*β* and TNF‐*α* mRNA expression. Because nuclear entry of the NF‐*κ*B p65 subunit is a prerequisite for transcriptional activation of proinflammatory genes [26], the suppressed p65 nuclear localization observed here matched the drop in proinflammatory cytokines. Bidirectional communication between the Keap1‐Nrf2 and NF‐*κ*B cascades is well documented [27, 28]. On the one hand, Nrf2 activation may attenuate upstream NF‐*κ*B–activating signals by scavenging ROS [29, 30]; on the other, p65 may competitively bind CBP/p300 or interact with Keap1 to repress Nrf2 activity [31, 32]. Our data are consistent with an inhibitory action of Nrf2 on NF‐*κ*B, although establishing causality will require epistatic experiments. Lee et al. [33] further showed that Keap1 promotes IKK*β* degradation through its E3 ubiquitin ligase activity, thereby inhibiting NF‐*κ*B activation. *Keap1* knockout might therefore in principle lead to IKK*β* accumulation and a proinflammatory outcome. Because we did not measure IKK*β* protein levels or phosphorylation status, the hierarchical relationship among Keap1, Nrf2, and NF‐*κ*B remains unresolved.

LPS‐induced inflammatory stress reproduces key features of the T2DM islet microenvironment, increasing *β*‐cell apoptosis and impairing proliferation [34]. When Keap1‐KO M2 macrophages were cocultured with LPS‐injured MIN6 cells, *β*‐cell viability rose and the apoptosis rate fell. Western blotting revealed higher Bcl‐2 together with lower Bax and cleaved‐caspase3, suggesting that Keap1‐KO M2 macrophages support *β*‐cell survival through paracrine mechanisms [35, 36]. The Keap1‐KO M2 group surpassed the WT M2 group not only in viability (*p* < 0.01) but also across antiapoptotic, anti‐inflammatory, and antioxidant indicators. This stronger protection at the population level was largely explained by the higher absolute number of M2 cells. *Keap1* thus appears to serve as an upstream regulator of M2 polarization efficiency, and its deficiency protects *β* cells at the population level by enlarging the M2 fraction.

In the Keap1 − KO M2 + MIN6 group, the proinflammatory cytokine IL‐1*α* and the lipid peroxidation product MDA decreased, whereas the antioxidant enzymes HO‐1 and SOD increased. Since HO‐1 and SOD were measured in MIN6 cells, this elevation reflects an enhanced antioxidant capacity of the *β* cells themselves. Within the islet microenvironment, ROS generated in response to cytokines can switch on inflammatory signaling and lock *β* cells into a self‐sustaining loop of injury [37]. By secreting anti‐inflammatory factors and paracrine signals, M2 macrophages may strengthen *β*‐cell antioxidant defenses and break this cycle [38], although the responsible mediators remain to be identified.

This study has three limitations. First, the cross‐species in vitro coculture of human THP‐1 and mouse MIN6 cells means the findings require validation in T2DM animal models. Second, experiments used only M2 polarization conditions, so the immunomodulatory effect of *Keap1* deficiency under M1 conditions was not tested. Third, the specific paracrine factors mediating *β*‐cell protection were not identified, and macrophage polarization stability was not monitored dynamically during coculture.

*Keap1* knockout promotes macrophage M2 polarization and attenuates LPS‐induced pancreatic *β*‐cell injury. Mechanistically, nuclear entry of NF‐*κ*B p65 is curtailed, transcription of proinflammatory cytokines falls, and Nrf2‐mediated antioxidant responses are probably strengthened. The Keap1‐KO M2 group showed stronger *β*‐cell protection than the WT M2 group, in line with the higher proportion of M2 cells caused by *Keap1* deficiency. This suggests that the protective effect mainly reflects expansion of the M2 population. Whether *Keap1* deficiency also enhances the capacity of individual M2 cells requires coculture with equal numbers of M2 cells to resolve. These findings clarify how macrophage *Keap1* shapes the islet inflammatory microenvironment. Its effects in vivo and under M1 polarization conditions remain to be determined.

## Author Contributions

**Baoliang Huang:** conceptualization, investigation, formal analysis, data curation, visualization, funding acquisition, writing — original draft. **Lunpan Mou:** investigation, validation, visualization. **Xiaoyan Huang:** investigation, data curation, validation. **Siyu Su:** methodology, investigation, formal analysis, resources. **Zhenzhen Hong:** methodology, supervision, project administration. **Yi Zhang:** conceptualization, supervision, project administration, funding acquisition, writing — review and editing.

## Funding

Funding for this work came from the Natural Science Foundation of Fujian Province (2023J011776), the Quanzhou High‐Level Talent Innovation and Entrepreneurship Project (2023C001YR), and the Fujian Normal University Qihang Fund (2025YJRHQH18).

## Disclosure

All authors have read and approved the final version of the manuscript. Yi Zhang, the corresponding author and manuscript guarantor, had full access to all of the data in this study and takes complete responsibility for the integrity of the data and the accuracy of the data analysis. The authors take full responsibility for the content of the published article.

## Ethics Statement

This study used only commercially available established cell lines—the human monocytic THP‐1 line (Wuhan Procell Life Science & Technology Co. Ltd., CM‐0233) and the mouse pancreatic *β*‐cell MIN6 line (Shanghai Fuheng Biotechnology Co. Ltd., FH0390). No human participants, human tissue samples, or identifiable human data were involved, and no live laboratory animals were used. On this basis, no ethical approval was required for this study.

## Conflicts of Interest

The authors declare no conflicts of interest.

## Data Availability

The raw data supporting this study are available from the corresponding author upon reasonable request.
